# Co‐Culture of *Lactobacillus bulgaricus* With *Streptococcus thermophilus* and *Bifidobacterium* Impact the Metabolism and Flavor of Fermented Milk

**DOI:** 10.1002/fsn3.70182

**Published:** 2025-05-06

**Authors:** Pingping Ma, Yanke Li, Jingjing Hao, Han Lu, Yannan He, Lihua Wei, Lianzhong Ai, Shijie Wang

**Affiliations:** ^1^ College of Food Science and Biology Hebei University of Science and Technology Shijiazhuang Hebei China; ^2^ Junlebao Dairy Co., LTD. Shijiazhuang Hebei China; ^3^ College of Health Science and Engineering University of Shanghai for Science and Technology Shanghai China

**Keywords:** *Bifidobacterium*, co‐culture, flavor substance, interaction, metabolomics

## Abstract

Incorporating *Bifidobacterium* into fermented milk alters the balance between *Lactobacillus bulgaricus* and *Streptococcus thermophilus* . We investigated the bacterial interaction and metabolism post‐fermentation and during 21‐day storage. Utilizing non‐targeted metabolomics and electronic nose technology, we assessed impacts on product quality and flavor. *Bifidobacterium* significantly increased the viability of the other two species, with AI‐2 levels rising in the mixed culture. Metabonomic analysis revealed distinct metabolic profiles, with *Bifidobacterium*‐fermented milk showing enriched key metabolites. Volatile compounds like ketones, aldehydes, esters, alcohols, and acids were identified, with 2‐heptanone and 2‐pentanone as initial discriminators and 2‐pentanone and acetaldehyde as key flavor compounds after storage. This study advances understanding of symbiotic interactions and metabolite profiles in fermented dairy ecosystems.

## Graphical abstract

The level of autoinducer‐2 (AI‐2) is maximized in dairy products fermented with *Bifidobacterium* species. *Bifidobacterium* significantly promoted the survival rate of *Lactobacillus bulgaricus* and *Streptococcus thermophilus* . The contents of caffeic acid and D‐galactose increased significantly after co‐culture. 2‐pentanone is the key flavor substance of three groups of fermented milk in two periods.

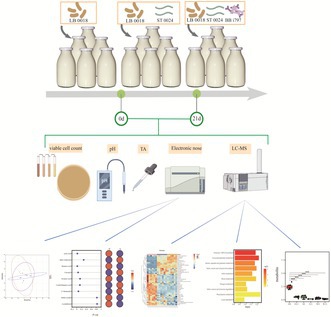

## 1 Introduction

*Bifidobacterium* exhibits ideal probiotic traits, making it a staple in fermented milk (Jena and Choudhury 2023). However, *Bifidobacterium* undergoes heterotypic fermentation, which not only produces lactic acid but also generates metabolites like acetic acid (Chen 2021). This can result in flavors that are unacceptable to consumers and may also alter the texture of the fermented milk (Li et al. 2020). Thus, incorporating *Bifidobacterium* into fermented milk results in a more complex fermentation process. The interaction between lactic acid bacteria (LAB) and *Bifidobacterium* is complex and subtle, involving metabolic complementarity, cell growth, and quorum sensing (QS) (Sarsan et al. 2021). For example, the oxygen consumption activity of *Streptococcus thermophilus* can create a favorable environment for the growth of anaerobic probiotics such as *Bifidobacterium* (Talwalkar and Kailasapathy 2004); co‐culture of *Bifidobacterium* with *S. thermophilus* can enhance the production of health‐promoting bioactive compounds, increase the production of beneficial metabolites (Linares et al. 2017), and promote the communication between gram‐positive bacterial cells through signal molecules (Meng et al. 2022).

The microbial metabolism of fermented milk has a decisive influence on its final product characteristics (Farag et al. 2020). For example, essential amino acids such as valine, isoleucine, leucine, and phenylalanine are transformed by peptidase produced by *Bifidobacterium* in yogurt production due to limited synthetic ability in the human body, thus improving the nutritional value of products (Li et al. 2021). Although studies have revealed the mechanism of the interaction matrix between *Lactobacillus bulgaricus* and *S. thermophilus* through metabolite analysis (Yang et al. 2024) and discussed the co‐culture system of *S. thermophilus*, *Bifidobacterium*, and *Lactobacillus plantarum* (Li et al. 2019), there is still a lack of in‐depth analysis on the interaction of microbial metabolites of *L. bulgaricus* , *S. thermophilus* , and *Bifidobacterium* after single culture and mixed culture.

Metabonomics is a powerful method that can reveal small molecular metabolites qualitative and quantitative changes during metabolism (Muthubharathi et al. 2021). Metabolomics techniques based on liquid chromatography‐mass spectrometry (LC–MS) have been successfully utilized across multiple disciplines, including food science (Zhong et al. 2022). It can reflect the state of cells in the milk matrix and provide insights into cell‐to‐cell communication. The accuracy and reliability of its ability to determine metabolites associated with microbial activities have been consistently validated (Naz et al. 2014). Metabonomics research helps to understand the influence of *Bifidobacterium* in fermented milk and guides the selection of starters. Electronic nose technology mimics the human sense of smell and is widely employed in the detection of dairy products (Yakubu et al. 2022). For example, Zoltan Kovac (Kovacs et al. 2020) used the electronic nose to monitor the aroma of fermented milk and distinguished yogurt samples fermented at different times according to their aroma components. Electronic nose analysis rapidly and accurately identifies flavor compounds, distinguishing between ketones, aldehydes, acids, and esters without contamination. This method has found extensive application in this field (Li, Wang, Zhao, et al. 2024).

Currently, *L. bulgaricus* and *S. thermophilus* are commonly used in fermented milk but do not confer intestinal benefits. This study aims to assess the production of metabolites, the dynamic changes in the metabolic profile, and the interactions between microbial strains following mixed fermentations with single and dual strains, as well as the addition of *Bifidobacterium*. Metabolites in fermented milk were analyzed using ultra‐performance liquid chromatography–tandem mass spectrometry (UPLC–MS/MS), and the data were processed using metabolomics. An electronic nose was employed to identify volatile flavor compounds in fermented milk. This study contributes to a deeper understanding of the interactions between fermented milk strains, particularly at the metabolic level.

## 2 Materials and Methods

### 2.1 Preparation of Fermented Milk

A 10% skimmed milk solution was prepared by dissolving skimmed milk powder, followed by the addition of 8% sugar. The mixture was hydrated for 30 min, heated to 60°C, homogenized, and sterilized at 95°C for 10 min. After cooling to 42°C, the mixture was inoculated with the following pre‐cultured strains: *L. bulgaricus* JMCC 0018, *S. thermophilus* JMCC 0024, and *Bifidobacterium* Ba i797 (provided by the JMCC Center of Junlebao et al.). *L. bulgaricus* JMCC 0018 was isolated from traditional fermented dairy products and pre‐cultured in MRS broth at 37°C under anaerobic conditions. *S. thermophilus* JMCC 0024, also isolated from traditional fermented dairy products, was pre‐cultured in MC broth at 37°C. *Bifidobacterium* Ba i797, isolated from infant feces, was pre‐cultured in TOS broth at 37°C under anaerobic conditions. All strains were cultured for three generations to ensure stable growth. The initial inoculation concentrations were 5 × 10^6^ CFU/mL for JMCC 0018 and Ba i797 and 1 × 10^6^ CFU/mL for JMCC 0024. Fermentation was monitored until the pH reached 4.5, at which point viable cell counts were measured, and the emulsion was disrupted. This process was repeated six times. Post‐demulsification, samples were stored at −80°C, except for those intended for 21‐day storage at 4°C. After storage, pH, viable cell counts, and autoinducer‐2(AI‐2) content were measured. Additionally, metabolomics and electronic nose analyses were performed on all samples.

### 2.2 Determination of pH, TA, and Viable Bacteria in Fermented Milk

The pH was determined using a pH meter. The phenolphthalein method determined the titration acidity with minor modifications (Zha et al. 2021). For sample preparation, 10 g of the sample was diluted with 20 mL of distilled water. After adding 2 mL of 0.5% phenolphthalein indicator, we titrated the mixture with a standardized sodium hydroxide solution until a light pink color appeared and remained stable for at least 30 s. The volume of sodium hydroxide added was recorded. The number of viable bacteria was determined using the dilution plate method. *S. thermophilus* was cultured in MC solid medium at 37°C for 48 h, *L. bulgaricus* was cultured in MRS solid medium for 48 h, and *Bifidobacterium* was cultured anaerobically in TOS medium for 72 h. After the culturing process was complete, the formed colonies were counted.

### 2.3 Detection of AI‐2

Dissolve 2 mL of fermented milk samples collected at two different time points in an equal volume of ethanol. Centrifuge the mixture to obtain the supernatant and dilute the DPD solution (0.3 mg/mL, dissolved in ultrapure water) to obtain a working standard solution with a concentration range of 10–7000 ng/mL. Prepare the DAN solution by dissolving 10 mg of DAN (Aladdin Inc., China) in 50 mL of 0.1 M HCl. Transfer 400 μL of the pretreated standard solution or supernatant into a 2‐L autosampler vial (Agilent Inc., USA), which contains an equal volume of DAN solution. Mix the two liquids thoroughly for 2 min. Incubate the samples in a water bath at 90°C for 40 min. After cooling, analyze the samples by HPLC–FLD. Inject 20 μL of the samples into a 1260 HPLC system (Agilent Inc., USA) equipped with a fluorescence detector for analysis. Separation was performed on an Agilent ZORBAX SB–C18 reversed‐phase chromatographic column (250 mm × 4.6 mm, 5 μm) maintained at 30°C. The mobile phase consisted of 0.1% formic acid (solvent A) and acetonitrile (solvent B), with a flow rate of 0.8 mL/min. The gradient elution curve was as follows: *t* = 0 min., 70% solvent A, 30% solvent B; *t* = 4 min., 70% solvent A, 30% solvent B; *t* = 12 min., 35% solvent A, 65% solvent B; *t* = 20 min., 35% solvent A, 65% solvent B; *t* = 24 min., 70% solvent A, 30% solvent B; *t* = 27 min., 70% solvent A, 30% solvent B. Then, the excitation wavelength and emission wavelength of the fluorescence detector were set to 271 nm and 503 nm, respectively.

### 2.4 Untargeted Metabolomics Analysis by LC–MS/MS

Some modifications were made based on the method described by Li et al. (2022). Fermented milk samples collected at 0 day and 21 days were thawed in an ice bath. Transfer 100 μL of sample into a 1.5 mL centrifuge tube, add 400 μL of extraction solvent (methanol:acetonitrile = 1:1, v/v) containing 0.02 mg/mL of internal standard (L‐2‐chlorophenylalanine), and shake at 1500 rpm at 10°C for 15 min (MSC‐100, Hangzhou All She). Place the sample at −20°C for 20 min, then centrifuge at 4°C at 18,000 *g* for 20 min (Microfuge 20r; Beckman Coulter Inc., Indianapolis, IN, USA). Take the supernatant, blow dry with nitrogen, and add 100 μL of mixed solution (acetonitrile:water = 1:1) for redissolution. Shake and mix at 1500 rpm at 10°C for 5 min, centrifuge for 10 min, and transfer the supernatant to an injection vial for analysis. The column used was an Acquity UPLC HSS T3 (100 mm × 2.1 mm I.D., 1.8 μm; Waters, Milford, USA). The UPLC instrument conditions were set as follows: Mobile phase A was 95% water + 5% acetonitrile (containing 0.1% formic acid), mobile phase B was 47.5% acetonitrile + 47.5% isopropanol + 5% water (containing 0.1% formic acid), column temperature was 40°C, flow rate was 0.4 mL/min, and injection volume was 10 μL. The MS conditions were set as follows: capillary temperature 325°C and heating temperature 425°C. We imported the original data into the metabonomics processing software Progenesis QI (Waters et al., USA) for tasks such as baseline filtering, peak identification, integration, retention time correction, peak alignment, etc., and ultimately obtained a data matrix containing information such as retention time, mass‐to‐charge ratio, and peak intensity. The software was then used to identify characteristic peaks, and the MS and MS/MS mass spectral information was matched against metabolic databases. The MS mass error was set to less than 10 ppm, and the matching score of the secondary mass spectra was used to identify metabolites. Public databases such as HMDB (http://www.hmdb.ca/) and METLIN (https://metlin.scripps.edu/) were utilized.

### 2.5 Electronic Noses to Determine the Change of Flavor Substances

Flavor analysis of fermented milk was performed using an electronic nose system (Heracles e‐nose, Alpha MOS, France). Following 21 days of fermentation and storage, 5 mL aliquots of each sample were transferred into 20 mL headspace vials for analysis. Prior to detection, frozen samples (−80°C) were equilibrated to room temperature. The electronic nose is calibrated before use. The analytical parameters were set as follows: injection volume, 5000 μL; incubation temperature, 50°C; injection speed, 125 μL/s; and inlet temperature, 200°C. The injection duration was 45 s, with a column temperature program starting at 0.5°C/s to 80°C, followed by a ramp of 3°C/s to 250°C, and a final hold at 250°C for 30 s. All experiments were conducted in quadruplicate.

### 2.6 Statistical Analysis

**FIGURE 1:**
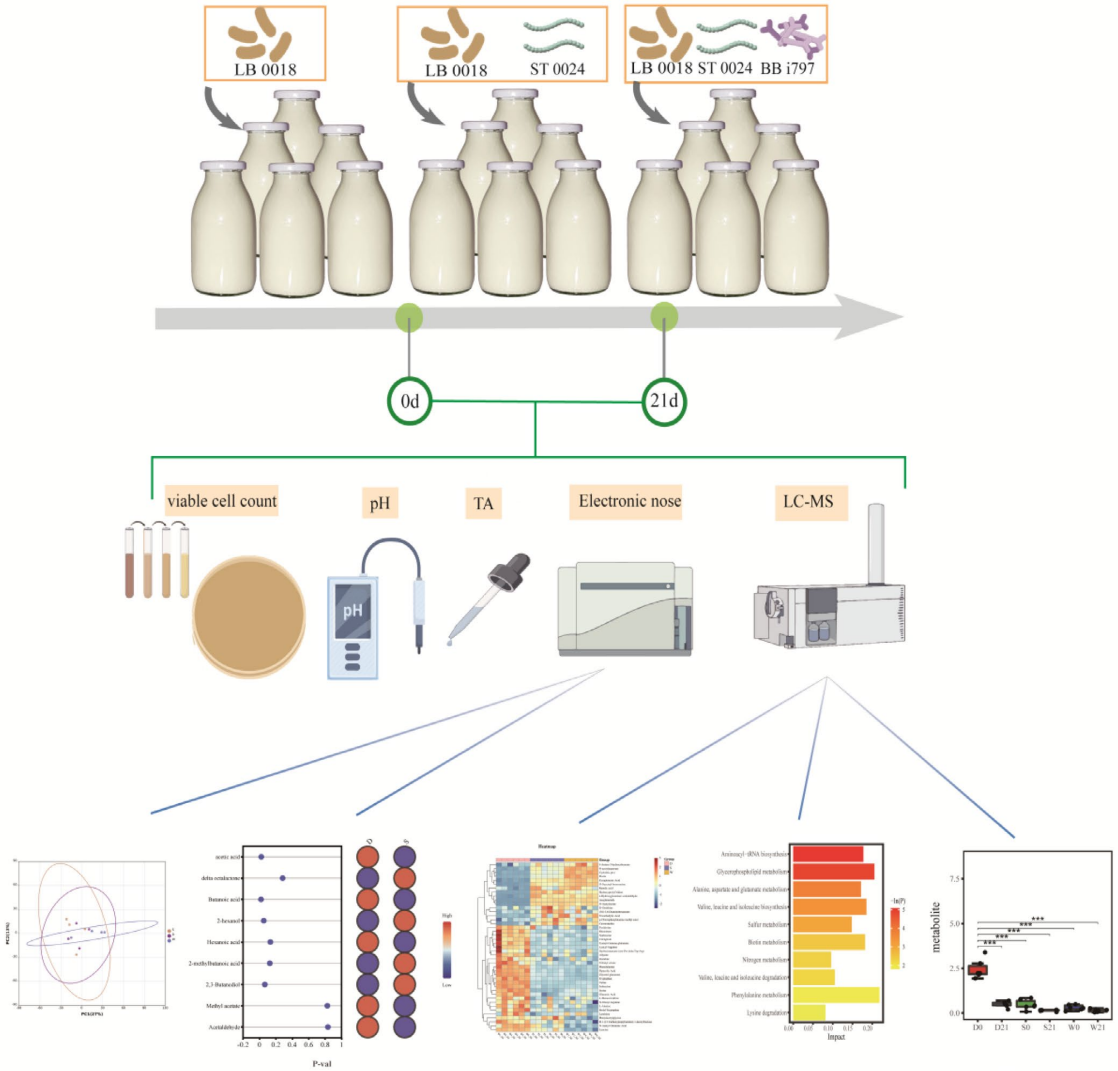
Experimental design for studying bacterial interactions in fermented milk. The experiment investigated the effects of *Streptococcus thermophilus* on *Lactobacillus bulgaricus* and the interaction mechanisms between *Bifidobacterium* and starter cultures. Three strain combinations were co‐fermented to produce fermented milk. Parameters including pH, titratable acidity (TA), viable cell counts, LC–MS‐based untargeted metabolomics, and volatile compound profiles were analyzed at the fermentation endpoint (0d) and after 21 days of storage (21d). Differential metabolites, metabolic pathways, and metabolic footprints were monitored to evaluate bacterial interactions and flavor development. (Figure created with Figdraw scientific illustration software).

GraphPad Prism 9.0.0 and R 4.2.3 were used to plot and process the experimental data. Metabolomics datasets were acquired from a minimum of six independent biological replicates (per experimental group) using LC–MS platforms, ensuring statistical power for detecting low‐abundance metabolites. Parallel analyses of volatile organic compounds (VOCs) using an electronic nose (e‐nose) were conducted with ≥ 4 independent replicates. Furthermore, MSDIAL was used for metabonomics data analysis, performing metabolite identification and accurate matching of the original data. The software carries out metabolite identification and accurate matching of the original data. Additionally, multiple regression analysis, including principal component analysis (PCA) and partial least squares discriminant analysis (PLS‐DA), was performed. Prior to conducting the Kruskal‐Wallis test, the normality of the data distribution was assessed using the Shapiro–Wilk test. The results indicated that the data did not follow a normal distribution (*p* < 0.05), justifying the use of non‐parametric tests. Raw metabolomics data were normalized using Pareto scaling to minimize technical variability, followed by log transformation to stabilize variance. For PLS‐DA model validation, a permutation test (1000 iterations) was performed to assess overfitting risk, with *R* ^2^ and *Q* ^2^ values reported in Figure S3. Peak detection parameters in Progenesis QI were set as follows: noise threshold = 5%, minimum peak width = 0.1 min, and retention time tolerance = 0.2 min. In addition, the Kyoto Encyclopedia of Genes and Genomes (KEGG) (https://www.genome.jp/kegg/) was used to analyze the pathways of related metabolites (Figure 1).

## 3 Results

### 3.1 Analysis of pH, TA, Viable Bacteria, and AI‐2 Content in Fermented Milk

**FIGURE 2:**
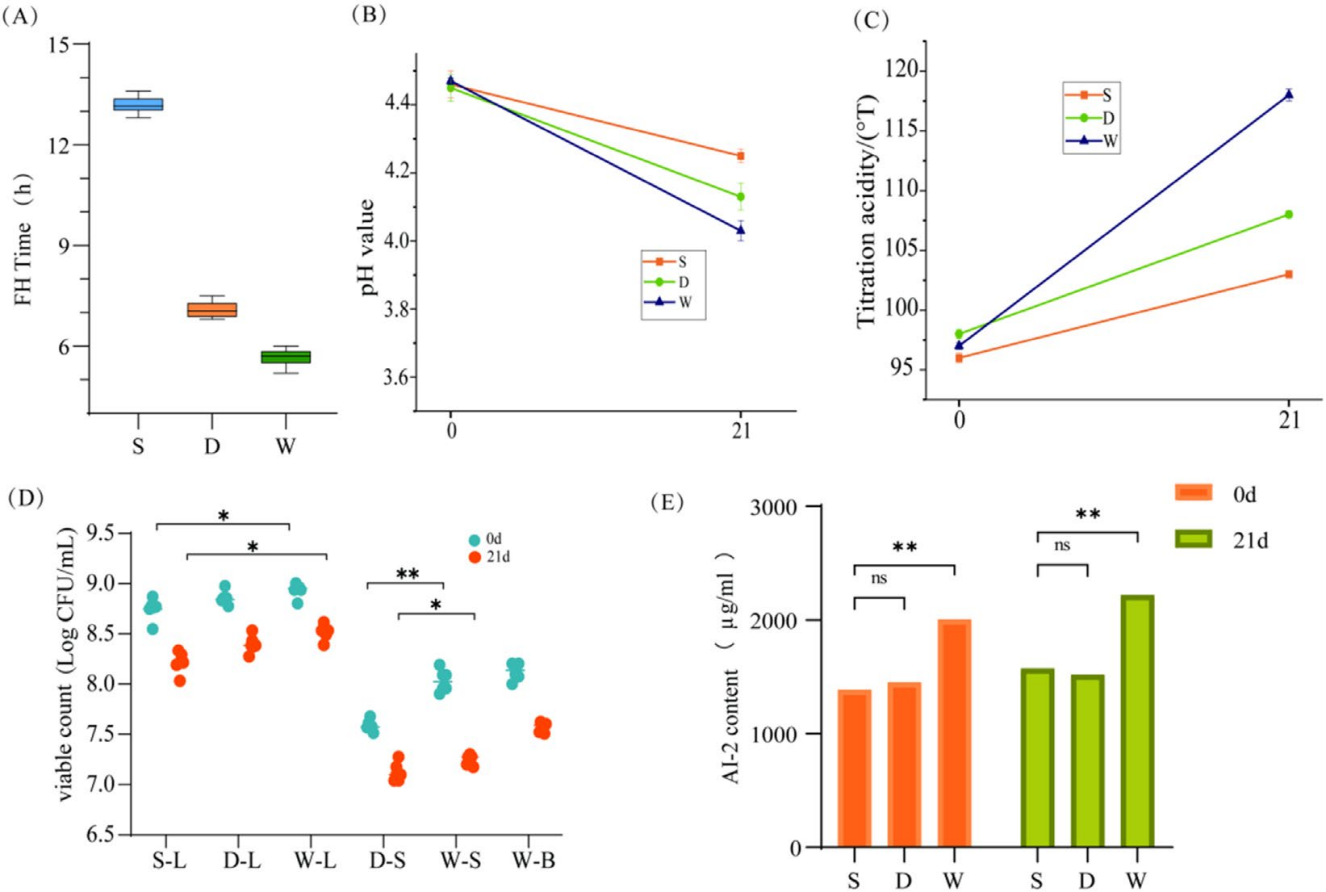
Fermentation characteristics and bacterial viability. (A) Fermentation time, (B) pH, (C) titratable acidity (TA), (D) viable cell counts, and (E) AI‐2 levels in three fermented milk groups at 0 day and 21 days. Data represent mean ± SD (*n* = 6). Statistical significance was determined by the Kruskal–Wallis test: *p < 0.05, **p < 0.01, ***p < 0.001. D‐S/W‐S, *S. thermophilus* counts in groups D/W; S‐L/D‐L/W‐L, *L. bulgaricus* counts in groups S/D/W; W‐B, *Bifidobacterium* counts in group W.

The time taken for fermentation completion (Figure 2A), pH values (Figure 2B), titratable acidity (TA, Figure 2C), and viable cell counts (Figure 2D) were measured for three groups of fermented milk at two different time points. The results showed that the addition of *S. thermophilus* alone or in combination with *Bifidobacterium* i797 accelerated the fermentation process. It took 13, 7, and 6 h for the pH of groups S, D, and W to reach 4.50, respectively. After 21 days of storage, the pH of groups S, D, and W decreased by 0.21 ± 0.2, 0.32 ± 0.2, and 0.44 ± 0.3, respectively. However, the TA of groups S, D, and W increased from 96 ± 0.4, 98 ± 0.4, and 97 ± 0.3 to 103 ± 0.2, 108 ± 0.3, and 118 ± 0.5, respectively. The addition of *Bifidobacterium* significantly boosted the growth rates of *L. bulgaricus* and *S. thermophilus* (*p* < 0.05). After 21 days, all groups maintained a high number of viable bacteria: *L. bulgaricus* decreased to 8.2–8.5 log CFU/mL, *S. thermophilus* to 7.1–7.3 log CFU/mL, and *Bifidobacterium* to 7.5–7.6 log CFU/mL. The results of AI‐2 showed no significant difference between groups S and D, but a significant difference was observed in group W compared with the other two groups.

### 3.2 Metabonomic Analysis of the Three Groups With Different Storage Times

**FIGURE 3:**
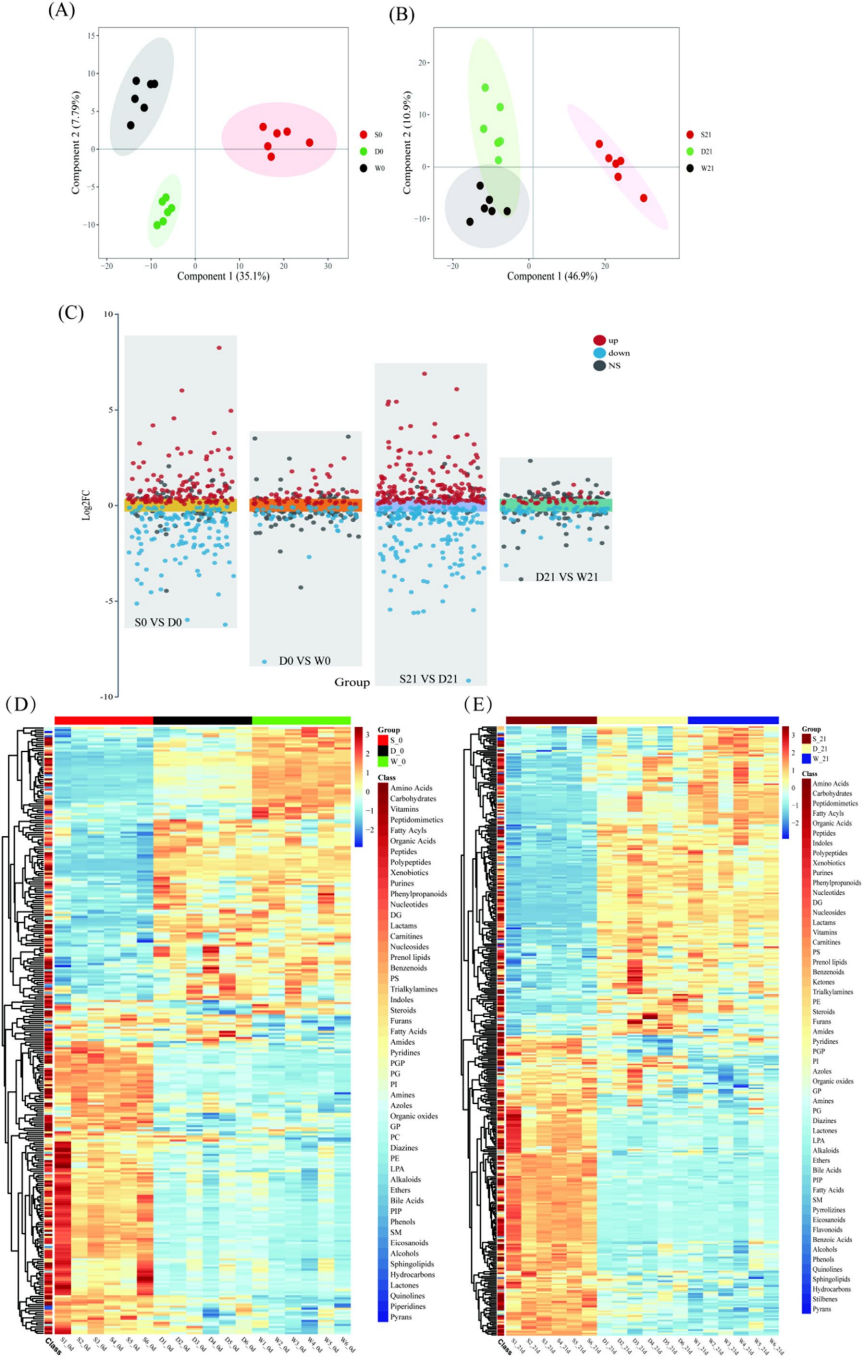
Multivariate analysis of metabolic profiles. PLS‐DA model results showing: PCA score plots for groups S/D/W at 0 day (A) and 21 days (B); (C) volcano plot of differentially abundant metabolites (red = upregulated, blue = downregulated, gray = nonsignificant; threshold: *p* < 0.05 and |log_2_FC| > 0); (D, E) heatmaps of metabolite profiles at 0 day (D) and 21 days (E). Color gradients reflect relative abundances (blue = low, red = high). Group clusters are indicated by colored bars.

**FIGURE 4:**
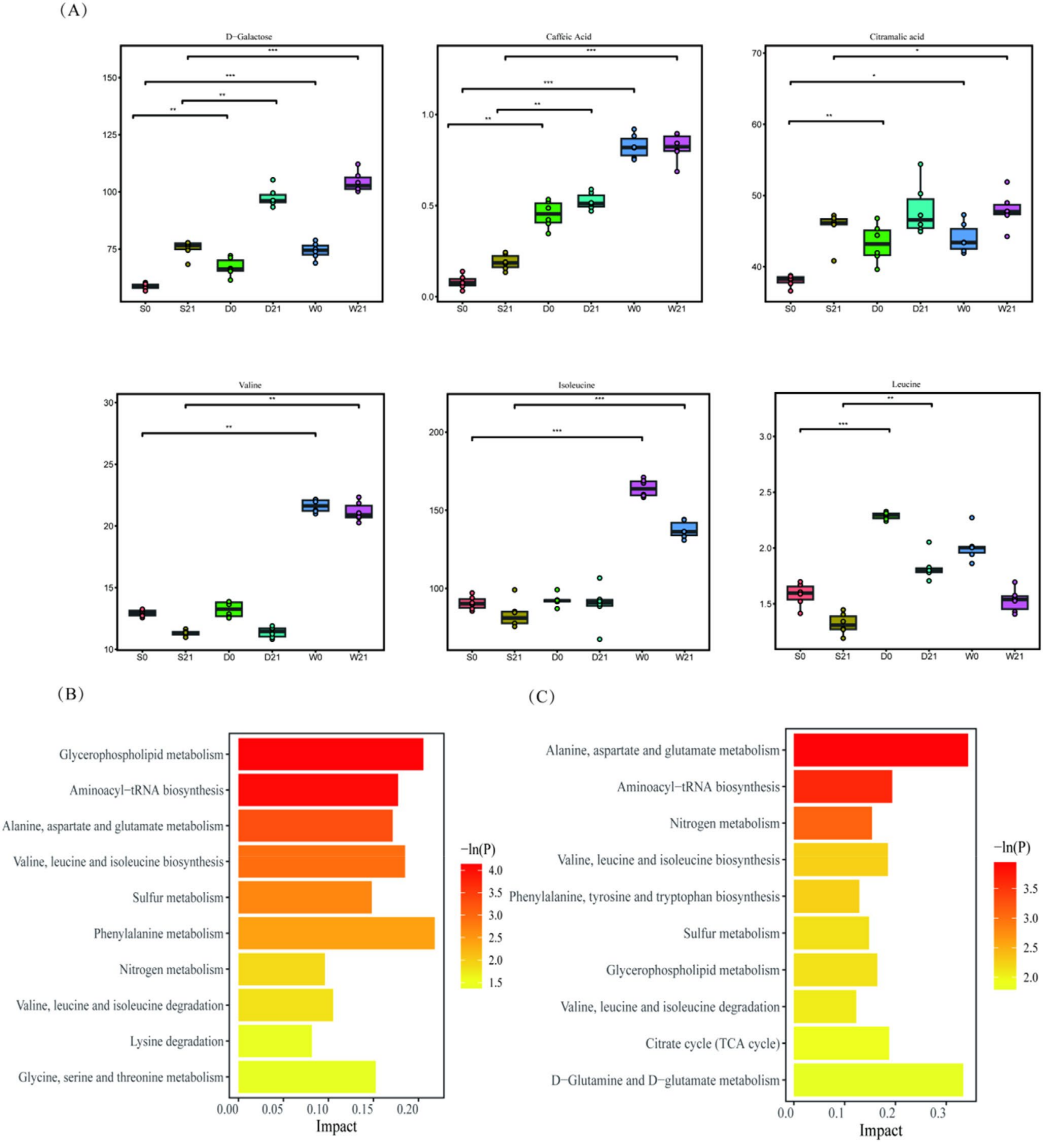
Key differential metabolites and pathway analysis. (A) Box plots of significantly altered metabolites among groups at identical storage times (Kruskal–Wallis test: *p < 0.05, **p < 0.01, ***p < 0.001). (B, C) Enriched metabolic pathways derived from differential metabolites at 0 day (B) and 21 day (C). Pathway significance is indicated by −ln(P).

To monitor technical variability and detect outliers, a multivariate control chart (MCC) based on principal component analysis (PCA) was generated by plotting the first principal component (PC1) scores against the injection sequence (Figure S4). All quality control (QC) samples (QC01–QC04) clustered tightly within ±2 standard deviations (SD), with experimental samples also predominantly distributed within ±2 SD and no outliers exceeding ±3 SD (dashed lines). These results confirm minimal technical drift and stable analytical performance. Additionally, Pearson correlation analysis between QC samples revealed near‐perfect pairwise correlations (range: 0.996–1; Figure S5), including 0.999 (QC01–QC02), 0.998 (QC01–QC03), and 0.997 (QC01–QC04). Such high correlations underscore minimal metabolomic variability among QCs, demonstrating rigorous experimental controls and high data reproducibility. PLS‐DA is a supervised learning technique used for classification and discriminant analysis, which builds models by maximizing inter‐class differences and minimizing intra‐class differences (Górski et al. 2016). Samples show apparent replicates clustering in the first and second principal components (Figure 3A,B), and samples from different groups are well separated. Based on the clustering pattern, the culture of fermented milk was identified as one of the factors influencing the overall metabolic structure. The Kruskal‐Wallis rank‐sum test was used to identify differential metabolites in different combinations at the same time point. Volcano plots (Figure 3C) were used to identify and visualize significant differences in metabolites among different groups of fermented milk. Compared to S0, D0 significantly upregulated 128 metabolites and downregulated 138 metabolites; compared to D0, W0 upregulated 128 metabolites and downregulated 138 metabolites; compared to S21, D21 upregulated 185 metabolites and downregulated 167 metabolites; compared to D21, W21 upregulated 35 metabolites and downregulated 59 metabolites. Next, heat maps of differentially expressed metabolites were generated, and hierarchical clustering analysis was performed to visualize changes in their relative levels (Figure 3D,E). The Random Forest algorithm (Figure S6) was used to perform a joint analysis of the metabolites, screening out key differentially expressed metabolites for each group and generating box plots (Figure 4). Key differential metabolites included D‐galactose, caffeic acid, citramalic acid, valine, isoleucine, and leucine. D‐galactose and caffeic acid increased significantly in groups D and W. Valine and isoleucine increased significantly in group W, while leucine increased significantly only in group D. Enrichment analysis of all differential metabolites revealed that glycerophospholipid metabolism was significantly enriched at 0 day, while alanine, aspartate, and glutamate metabolism were significantly enriched at 21 days. CDP‐diacylglycerol was both upregulated and downregulated in the pathway at 0 day, and L‐alanine, L‐glutamine, L‐glutamate, and N‐acetyl‐L‐aspartate changed at 21 days (Figure S6). The second most significant metabolic pathway at both 0 day and 21 days was aminoacyl‐tRNA biosynthesis.

### 3.3 Electronic Nose Analysis

**FIGURE 5:**
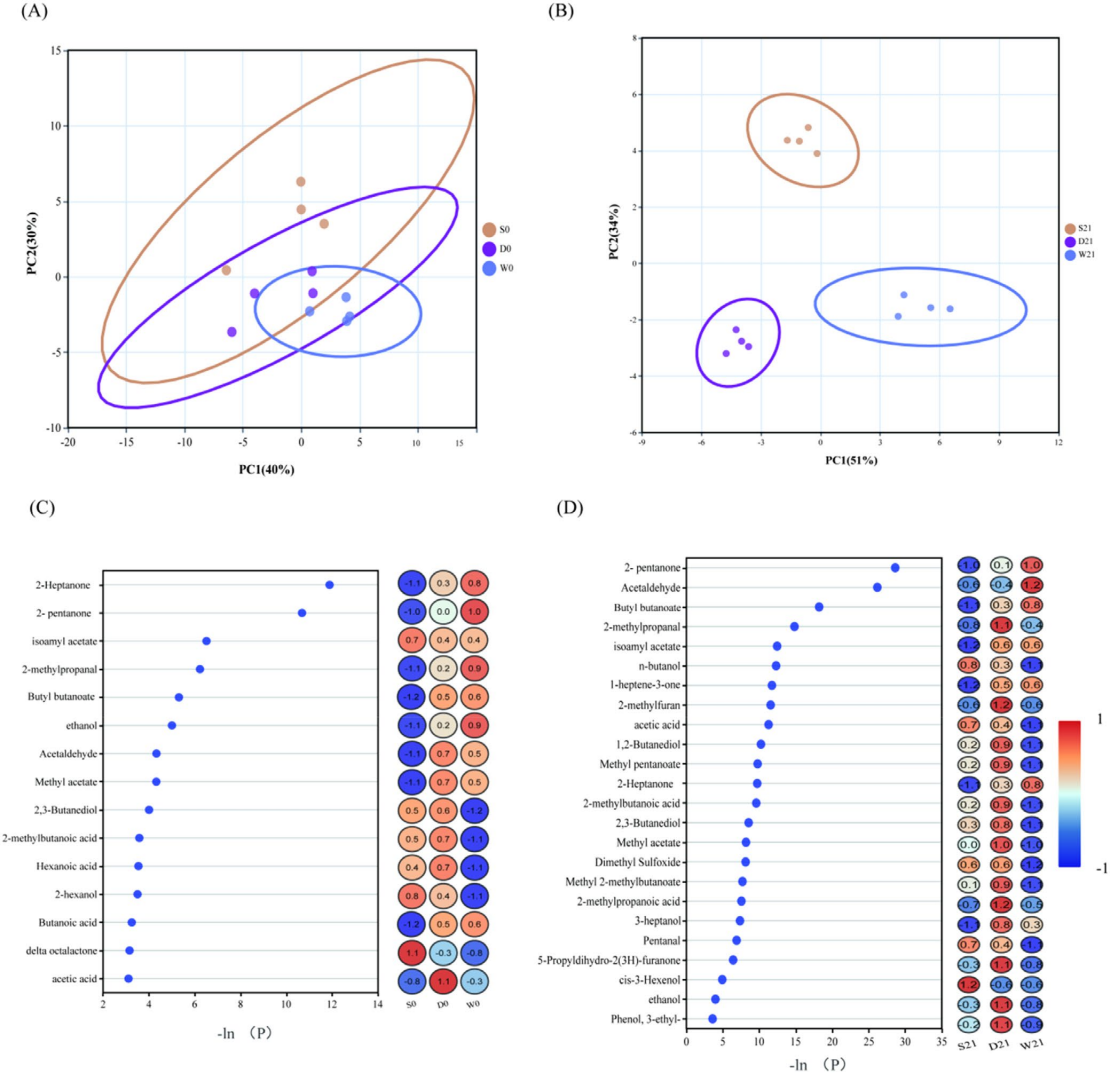
Electronic nose analysis of volatile compounds. PCA score plots distinguish volatile profiles among groups at 0 day (A) and 21 days (B). (C, D) Heatmaps showing relative abundances of volatile compounds at 0 day (C) and 21 days (D). Color intensity represents −ln(P) for significance (red = high, blue = low). Group clustering is denoted by colored sidebars.

Electronic nose PCA analysis showed that there was a certain degree of overlap in the scores of principal components of flavor substances among the S0, D0, and W0 groups, which may indicate that the volatile substances between these groups were similar (Figure 5A). However, the principal components of fermented milk in each group could be well separated after 21 days of storage (Figure 5B). Differential compounds were selected to create a heatmap, and compounds with significant differences (*p* < 0.05) were identified (Figure 5C,D). These compounds included ketones, aldehydes, esters, alcohols, and acids, which are typical components of most dairy products. The results showed that the contents of 2‐heptanone, 2‐pentanone, 2‐methylpropanal, butyl butyrate, ethanol, butanoic acid, and acetaldehyde increased significantly after adding *S. thermophilus* and *Bifidobacterium* on day 0. After adding *Bifidobacterium*, the contents of 2,3‐butanediol, 2‐methylbutanoic acid, hexanoic acid, and 2‐hexanoic acid decreased significantly. Notably, the acetic acid content increased significantly after the addition of *S. thermophilus* but decreased significantly after adding *Bifidobacterium*. On day 21, after adding *S. thermophilus* and *Bifidobacterium*, the contents of 2‐pentanone, acetaldehyde, butyl acetate, and isoamyl acetate in Groups D and W increased significantly, while the contents of n‐butanol, acetic acid, 1,2‐butanediol, methyl pentanoate, 2‐methylbutanoic acid, and 2,3‐butanediol decreased significantly, such as n‐butanol, acetic acid, 1,2‐butanediol, methyl pentanoate, 2‐methylbutanolic acid, 2,3‐butanediol, etc.

## 4 Discussion

This study discussed the effects of different strain combinations on the quality of fermented milk. The results showed that double and triple strains significantly shortened the fermentation time and caused a decline in pH compared to single‐strain fermentation. This was consistent with other research (Sun et al. 2024). After 21 days of storage, the pH value of the three groups of fermented milk decreased significantly, and the titrated acidity increased significantly, which may reflect the continuous fermentation of bacteria and the accumulation of lactic acid (Li et al. 2019). Additionally, *Bifidobacterium* significantly increased the viability of *L. bulgaricus* and *S. thermophilus* . It may be due to *Bifidobacterium* releasing growth factors or metabolites into the milk (Sun, Guo, et al. 2023). These promoted the growth of *Lactobacillus* and *Streptococcus*. Studies have shown that phenylalanine may promote the proliferation of LAB (Castillo et al. 2022), and the results of this study showed that the content of phenylalanine increased significantly after adding *Bifidobacterium*, which may be that the increase in phenylalanine content further promoting the proliferation of LAB. QS constitutes a cell density‐dependent communication protocol in microorganisms, enabling the exchange of information and interaction among them through a variety of signaling molecules. This mechanism is pivotal for the coordination of microbial activities and interspecies interactions. AI‐2 regulates the communication among lactic acid bacteria and their populations. AI‐2 is the only signaling molecule identified to date that is recognized by both gram‐positive and gram‐negative bacteria. Our research results have shown that adding *Bifidobacterium* can significantly increase the content of AI‐2, suggesting that *Bifidobacterium* can promote communication between bacteria.

Metabonomics analysis employs high‐throughput techniques to quantify small‐molecule metabolites within biological systems, providing a dynamic snapshot of metabolic phenotypes. In fermented milk studies, this approach reveals not only quality attributes (e.g., organic acid profiles) but also functional characteristics mediated by microbial activity, such as bioactive peptide generation (Górski et al. 2016). As a supervised multivariate method, PLS‐DA addresses the “curse of dimensionality” in metabolomics data by projecting high‐dimensional spectral features (X‐matrix) onto latent variables (LVs) that maximize covariance with predefined class labels (Y‐matrix). However, this discriminative power comes at interpretability costs: LV complexity escalates with component count, obscuring individual metabolite contributions. Overfitting risks necessitate rigorous validation. In our study, PLS‐DA differentiated post‐fermentation and 21‐day storage metabolites (Figure 3). Key VIP metabolites (VIP > 1) were found (Wang et al. 2006). As an ensemble tree‐based algorithm, RF mitigates single‐tree overfitting by aggregating predictions from multiple decorrelated trees. For some samples and some metabolites, bootstrap sampling generates subsets. Predict via majority vote (classification) or averaging (regression). Advantages critical for metabolomics include handling non‐linear relationships, quantifying metabolite importance via the Gini index or permutation. Limitations observed: suboptimal for low‐abundance metabolites (mean decrease in accuracy < 0.01), Tendency to prioritize high‐variance features (Moradi et al. 2020). While PLS‐DA excels in dimension reduction and hypothesis generation, RF provides robust classification and feature ranking. Their consensus on core metabolites strengthens biological validity, whereas discrepancies may reflect PLS‐DA's sensitivity to covariance structures versus RF's non‐linear feature weighting (Paul et al. 2018). Future studies could integrate pathway‐centric models (e.g., MBRole) to bridge statistical findings with metabolic network dynamics. Through thermography and random forest analysis, some key differential metabolites that may play a key role in different strains were screened out. For example, as nitrogen sources, amino acids triggered a series of biochemical reactions and promoted the optimal growth state by regulating microbial communities (Zengler and Zaramela 2018). This study identified D‐galactose and caffeic acid as critical differential metabolites that may be involved in the interaction between lactic acid bacteria. The relative content of caffeic acid increased significantly with the increase of probiotics, which may enhance the antibacterial and antioxidant activities of fermented milk, consistent with findings that phenolic compounds like caffeic acid contribute to the functional properties of fermented dairy products (Wu et al. 2021). Furthermore, the observed increase in D‐galactose content when *S. thermophilus* and *Bifidobacterium* were co‐cultured can be attributed to the metabolic synergy between these strains. Specifically, *S. thermophilus* preferentially metabolizes glucose from lactose, releasing galactose into the milk matrix (Li, Wang, et al. 2023). In addition, the metabolites of co‐culture *Bifidobacterium* and lactic acid bacteria, such as valine and isoleucine, also show their importance in the process of compound fermentation (Wang et al. 2021; Sun, Yu, et al. 2023; Huang et al. 2022). Some research reported that several amino acids, such as arginine, isoleucine, leucine, tryptophan, tyrosine, cysteine, and valine, are stimulatory or essential for the growth of *Bifidobacteria* (Liu et al. 2021). The combined action of lactic acid bacteria and *Bifidobacterium* promotes fat and carbohydrate metabolism and accelerates the production of amino acids and peptides (Zhang et al. 2020). Especially hydrophobic amino acids, such as valine, have antihypertensive biological activity (Yuan et al. 2022), valine and arginine, as precursors of α‐ketoglutarate, play a vital role in the TCA cycle (Ao et al. 2023). The biosynthesis of phenylalanine, valine, leucine, and isoleucine and the changes in tyrosine metabolism may affect the strain's growth, acid production, and protein hydrolysis (D'Este et al. 2018). Notably, the reduction of isoleucine contributes to the production of 2‐methylbutyric acid and 2‐methylpropionic acid (Yu et al. 2024). The quality and flavor characteristics of fermented milk are also influenced by cell quorum sensing systems, such as the biosynthesis of leucine and isoleucine and the production of ketone flavor substances, which are controlled by quorum sensing (QS) (Shi et al. 2024; Guo et al. 2024; Siragusa et al. 2011). These results not only align with existing literature but also provide novel insights into the metabolic pathways and interactions driving strain‐specific fermentation outcomes, advancing our understanding of microbial contributions to dairy product functionality and sensory attributes.

The glycerol phospholipid metabolism pathway plays a crucial role in resisting ethanol toxicity. CDP‐diacylglycerol can be converted into phosphatidylethanolamine, which promotes cell membrane stability, signal transduction, fat metabolism, and cardiovascular health (Hao et al. 2023). On the other hand, the alanine, aspartate, and glutamate metabolism pathways explain the differences in volatile flavor compounds among samples. L‐alanine, L‐glutamine, and L‐glutamate are essential in the alanine, aspartate, and glutamate metabolism (Guo et al. 2024). They interconvert through transamination, which helps maintain amino acid balance and supports other biosynthetic pathways. Wu et al. (2024) also observed significant enrichment of glutamate and tyrosine metabolism pathways during storage, which accounts for the high viable bacterial counts during this period.

Electronic nose PCA analysis revealed distinct temporal shifts in the main components of fermented milk during 21‐day storage. While initial compositions (Day 0) were similar across groups, significant changes emerged by Day 21, particularly in ketones (e.g., 2‐heptanone, 2‐pentanone) and acids (e.g., 2‐methylbutanoic acid, hexanoic acid). The co‐culture of *S. thermophilus* and *Bifidobacterium* drove these changes, with galactose accumulation enhancing sweetness and masking sourness via Maillard reactions (Li, Wang, et al. 2023; Pan et al. 2014). Notably, the addition of *Bifidobacterium* markedly increased ethanol content, aligning with prior reports on galactose metabolism (Li, Wang, et al. 2023; Zhao, Tang, et al. 2023). Ketones, key contributors to creamy and fruity notes (e.g., 2‐heptanone, 2‐pentanone), are derived from lipid oxidation and amino acid degradation (Wu et al. 2024, 2023; Liu et al. 2022). Our results corroborate their role in flavor enhancement, as co‐cultured samples exhibited pronounced creamy aromas. However, the involvement of caffeic acid in ketone production remains contentious. While we observed its potential modulation of unsaturated fatty acid metabolism (Wu et al. 2021), other studies detected caffeic acid in fermented dairy products without direct links to volatile flavor changes (Zhao, Tang, et al. 2023). It is postulated that the disputed function of caffeic acid in flavor regulation might be attributed to the strain‐specific metabolic interplay. This discrepancy may stem from strain‐specific metabolic interactions or analytical sensitivity differences (e.g., electronic nose vs. GC–MS) (Kovacs et al. 2020; Zhao et al. 2024)., underscoring the need for multi‐method validation in future work. Branched‐chain amino acids (BCAAs: valine, isoleucine, and leucine) served as critical precursors for ketone formation. Their enzymatic conversion to ketoacids likely underpinned the observed increases in 2‐heptanone and 2‐pentanone (Li, Wang, Zheng, et al. 2024; Wu et al. 2023). Conversely, phenylalanine degradation generated bitter‐tasting compounds (e.g., phenylacetic acid) (Fu et al. 2022; Li, Gao, et al. 2023; Qiu et al. 2022), while γ‐glutamyltransferase activity from glutamate contributed umami notes (Xia et al. 2021; Zhao, Zheng, et al. 2023). Lipid metabolism further shaped flavor profiles, with phospholipid oxidation releasing aldehydes (e.g., acetaldehyde) and esters (e.g., isoamyl acetate) linked to fruity and sweet aromas (Liu et al. 2022; Fu et al. 2022; De Cadiñanos et al. 2019). Notably, β‐galactosidase activity from *Bifidobacterium* likely elevated acetaldehyde levels—a dynamic rarely achieved in single‐strain fermentations (Zhao, Tang, et al. 2023; Mituniewicz‐Małek et al. 2017).

Our findings partially align with the literature on flavor biochemistry but highlight key divergences. For instance, while lipid‐derived volatiles (e.g., butyl butyrate) are widely recognized as flavor determinants (De Cadiñanos et al. 2019; Tian et al. 2023; Dinh et al. 2021), their abundance in our system exceeded typical ranges reported for single‐strain fermentations (Wang et al. 2021). This may reflect synergistic microbial interactions in co‐cultures, enhancing pathway cross‐talk. Similarly, the controversial role of caffeic acid suggests context‐dependent effects, possibly mediated by microbial community structure or substrate availability. To resolve these ambiguities, integrated multi‐omics approaches (e.g., metabolomics‐transcriptomics) are essential to map strain‐specific contributions and pathway regulation. Additionally, targeted investigations into indirect flavor modulation mechanisms (e.g., microbial co‐metabolism of phenolic acids) could clarify compounds like caffeic acid's dual roles as metabolic substrates versus flavor modulators (Chen et al. 2023; Fang et al. 2020).

This study investigated the influence of different strain combinations on the quality of fermented milk, revealed the metabolic characteristics and production of flavor substances of the culture through the metabolome and electronic nose, and screened out the key differential metabolites in different strain combinations and storage periods. Future prospects might include a multi‐omics integration or a deeper dive into the molecular mechanisms underlying quorum sensing and interspecies metabolic signaling, which would further strengthen the impact of the study, including a more in‐depth analysis of microbial community structure, metabolites, and flavor properties during fermentation. Furthermore, future studies could also investigate the effects of different strain combinations on the nutritional value and health benefits of fermented milk and how to improve the quality and taste of the products by optimizing the fermentation conditions.

## 5 Conclusions

The study concluded that the combination of bacterial strains in fermented milk significantly impacts its quality and flavor profile. The use of double and triple strains accelerates fermentation, reduces pH, increases AI‐2 content, and concludes the production of key metabolites such as D‐galactose and caffeic acid, which are critical for flavor development and bacterial growth. The introduction of *Bifidobacterium* promotes the viability of other lactic acid bacteria, enhancing the fermentation process and flavor complexity. Metabonomics analysis and electronic nose technique reveal the dynamic changes in metabolites and aroma compounds during fermentation and storage, highlighting the importance of strain interactions and metabolic pathways in shaping the characteristics of the final product. Future research could expand on these findings to optimize fermentation conditions for improved nutritional value, health benefits, and sensory qualities of fermented milk.

## Author Contributions

**Pingping Ma:** data curation (equal), methodology (equal), software (equal), writing – original draft (equal). **Yanke Li:** methodology (equal), software (equal), writing – review and editing (equal). **Jingjing Hao:** formal analysis (equal), software (equal), writing – review and editing (equal). **Han Lu:** data curation (equal), software (equal), writing – review and editing (equal). **Yannan He:** validation (equal), writing – review and editing (equal). **Lihua Wei:** project administration (equal), writing – review and editing (equal). **Lianzhong Ai:** formal analysis (equal), supervision (equal), writing – review and editing (equal). **Shijie Wang:** funding acquisition (equal), supervision (equal), writing – review and editing (equal).

## Ethics Statement

The strains employed in this study are the exclusive property of Junlebao Dairy Group Co. Ltd. The utilization of these strains has been duly approved by Junlebao Company. Moreover, researchers affiliated with Junlebao actively participated in this study. All related research endeavors fully comply with ethical standards and are free from any disputes.

## Conflicts of Interest

The authors declare no conflicts of interest.

## Supporting information


Data S1.

## Data Availability

The original data is provided with the attachment.
