# Supplementary material for: Co‐Culture of Lactobacillus bulgaricus With Streptococcus thermophilus and Bifidobacterium Impact the Metabolism and Flavor of Fermented Milk
Source: Food Sci Nutr. 2025 May 6;13(5):e70182. doi: 10.1002/fsn3.70182 (PMC12055522; doi:10.1002/fsn3.70182)
Supplement: Supplementary file 1 — Data S1. [file FSN3-13-e70182-s001.docx]

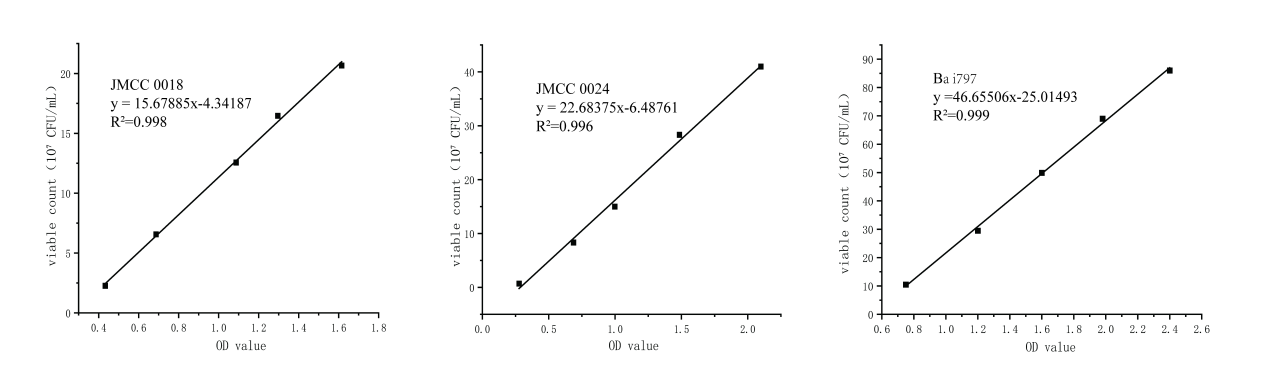


Supplementary Figure 1: The growth curve graph of the three strains, where the horizontal axis represents the OD value, and the vertical axis represents the viable cell count.


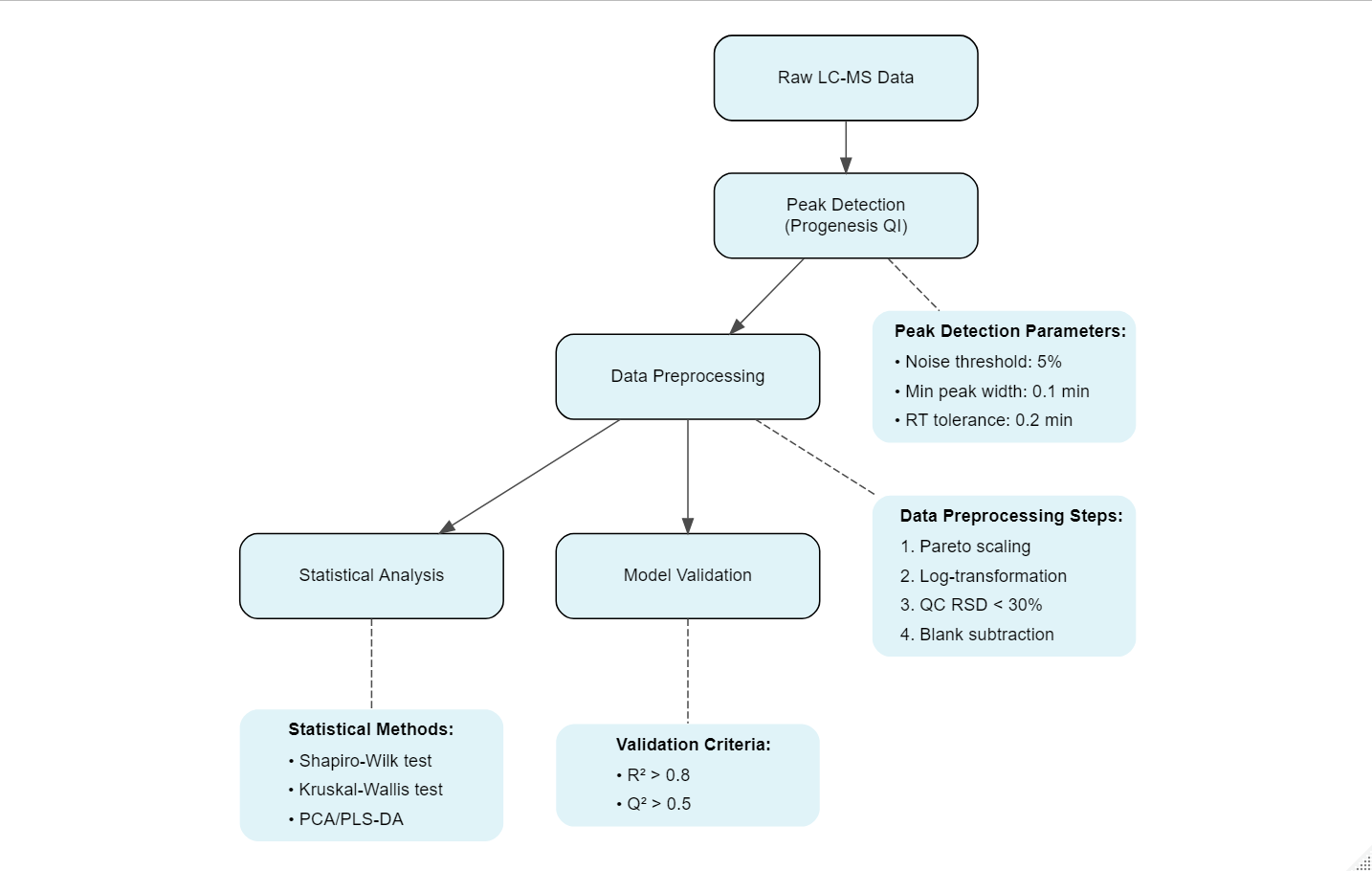


Supplementary Figure 2: Workflow diagram of LC-MS data processing.

Supplementary Figure 3; PLS-DA permutation test analysis of fermented milk at Day 0 (A) showed R²Y = 0.966 and Q²Y = 0.718, while at Day 21 (B), values were R²Y = 0.959 and Q²Y = 0.842. Permutations: n=1000; significance threshold defined as Q²Y > 95% of the permutation null distribution (empirical p<0.05). Vertical axis indicates the correlation strength between variables and the PLS-DA model (range: 0.0–0.9). Gray shaded area/dashed line represents the 95% confidence interval of the permutation-generated null distribution; red vertical line marks the observed Q²Y value, significantly exceeding random permutation results.
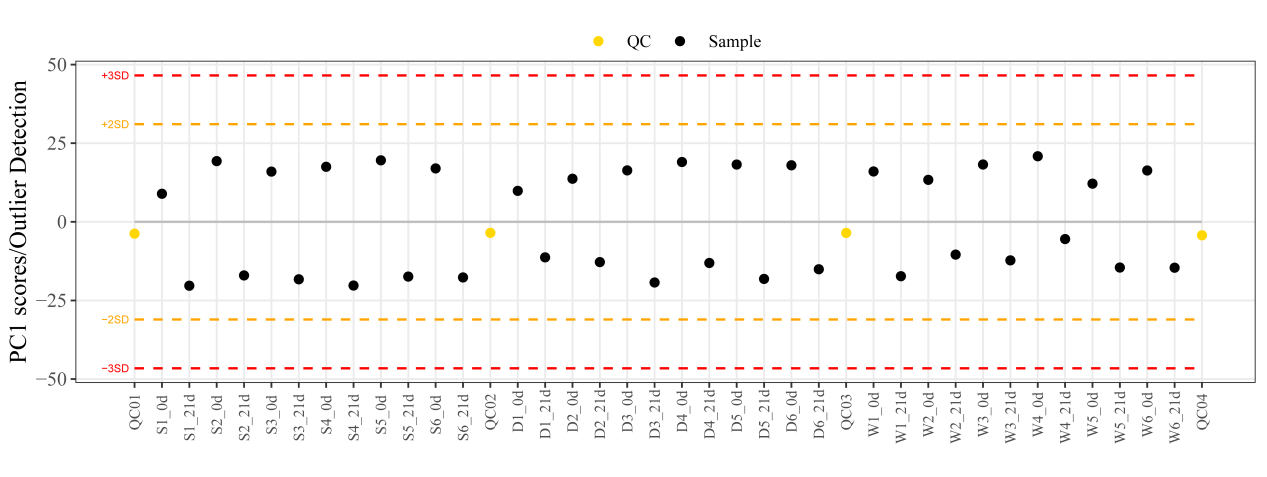


Supplementary Figure 4: Multivariate Control Chart (MCC) of PC1 scores across the injection sequence PC1 scores (y-axis) for QC samples (QC01–QC04, labeled) and experimental samples are plotted against the injection order (x-axis). Dashed lines represent ±2SD and ±3SD control limits. Tight clustering of QCs and samples within ±2SD confirms analytical stability.


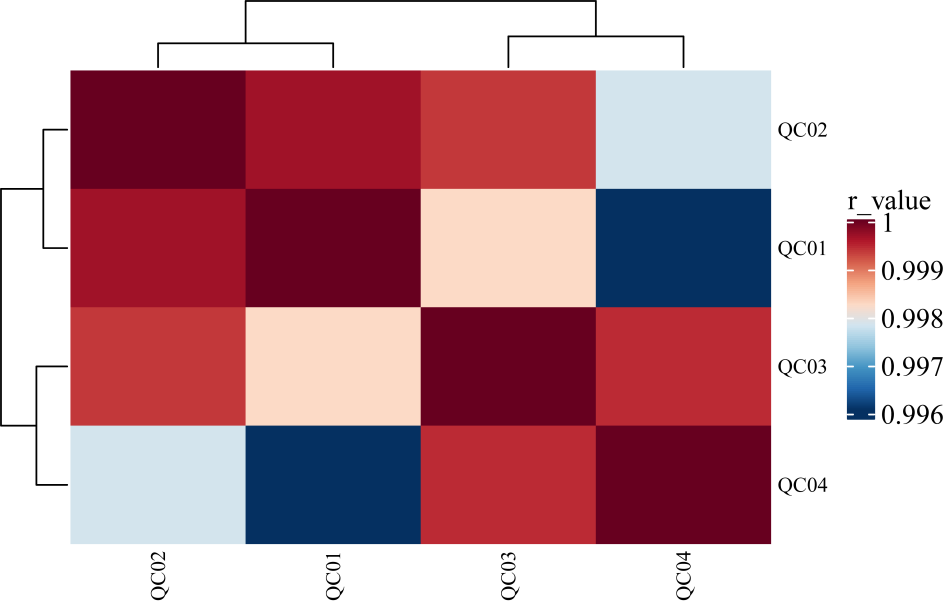


Supplementary Figure 5: Correlation between QC samples is shown in the Heatmap. The color of each cell represents correlation coefficient between QC samples. Correlation coefficient near to 1 indicates that the quality control is good and the data is reliable.


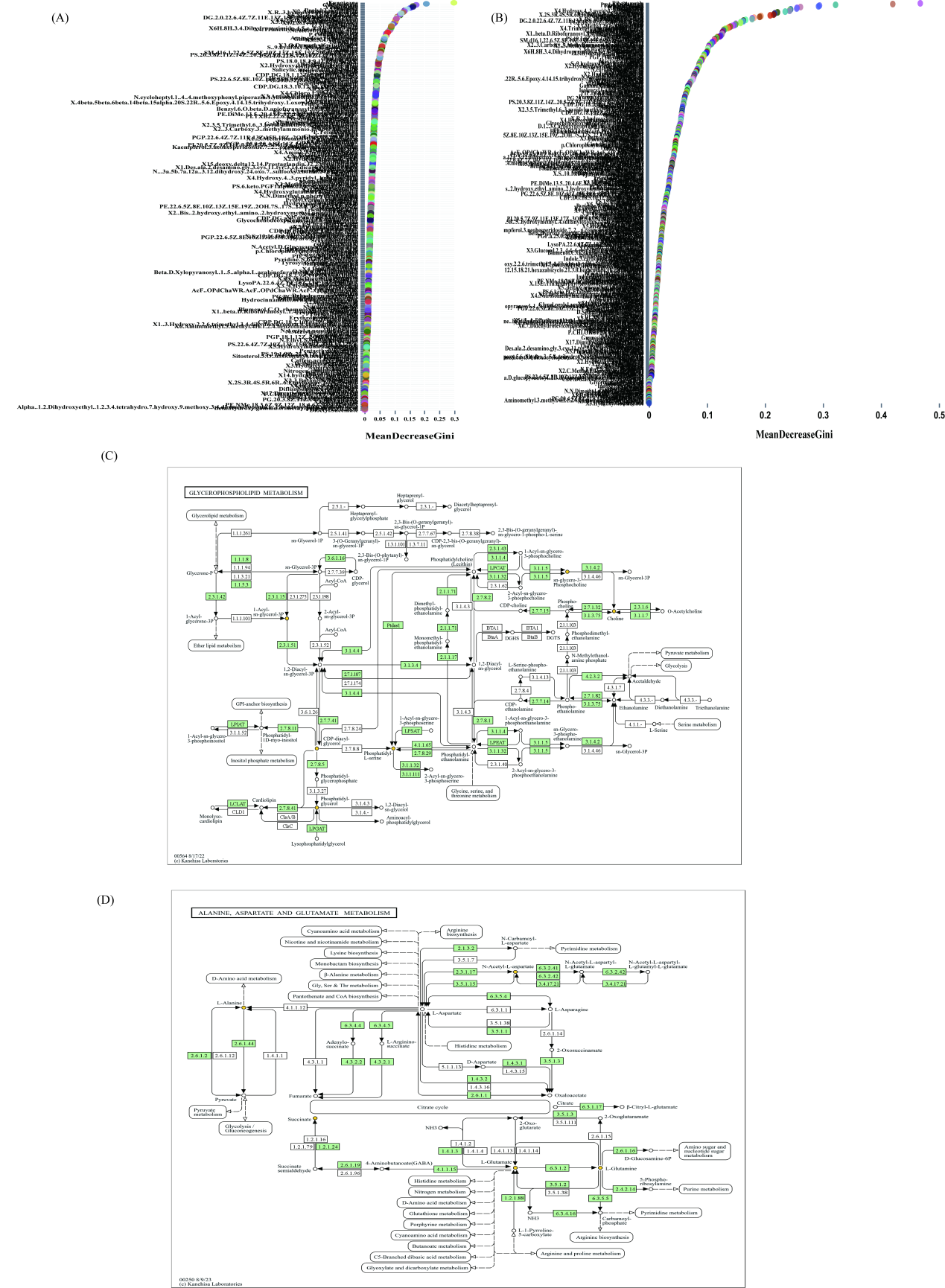


Supplementary Figure 6: Random forest map of three groups of fermented milk , and the top 50 of Mean Reduction were selected to screen key differential metabolites(A) and (B),and figure shows KEGG pathway diagrams, namely S0 vs D0 vs W0 (C), and S21 vs D21 vs W21 (D), where C represent glycerophospholipid metabolism, and D represents alanine. Metabolism of aspartate and glutamine; the circle pointed to by the arrow in the figure represents metabolites; red refers to up-regulation, blue refers to down-regulation, and yellow refers to both up-regulation and down-regulation.
